# Annotations

**Published:** 1894-09-22

**Authors:** 


### Annotations.

### A New Cure: the Ass to the Rescue.

Another cure for tuberculosis is announced. Three years ago tuberculin was the cure; two years ago it was goat's serum; to-day it is the serum of asses. According to the report published, Dr. Viquerat, of Geneva, has for some time been labouring to discover a "cure" for consumption, and at last he has been enabled to announce results which are said to be "extraordinary." Twenty-seven tuberculous patients "of the second or third degree" have undergone treatment, and twenty-five of them are declared to be entirely cured. Dr. Viquerat has explained his method in a "public lecture," at which "a number of doctors were present." This is the first false note which strikes upon the medical ear. A public lecture is not the occasion, an indiscriminate lay and medical audience is not the tribunal for the judicial consideration of new methods of treating tuberculosis. A man must be judged by his methods as well as by the results he claims. What other methods could Dr. Viquerat have employed if he had been the veriest "quack" instead of a regular practitioner? It is sometimes said that medical prejudice is so strong that the man who has a new cure, unless he be a person whose reputation is already established, is obliged to appeal to the public. This is perfect nonsense. We are not concerned to deny that medical men have their prejudices. In this respect they are exactly like other people. But if the least known member of the profession were to discover a real cure for consumption, he would at once become the best known, and the profession itself would be the first to proclaim his merits. But then the "cure" must be a "real cure." At the present stage there is no call to discuss Dr. Viquerat's claims. If his cure should prove to be genuine, his methods will be forgiven, even by the most exigent defenders of professional orthodoxy. But for the moment the "boom" has the appearance of being commercial rather than scientific or humane.

### Undignified French Dignities.

The French Academy of Medicine is ordinarily a dignified body. It must have met after dinner the other day, if the report of the *Times* as to its pronouncement on bicycling be even approximately correct. Not only did the members of the Academy unanimously put the cart before the horse in passing a resolution at the commencement instead of at the close of a discussion on cycling, but the cart thus dislocated was of so peculiar a kind that one of the Paris evening papers was constrained to call attention to its ridiculousness by a very uncomplimentary reference to professional fees. Thus says the *Times*: "Upon the proposal of Dr. Verneuil," after some desultory talk upon the subject, "it was decided that a full inquiry should be opened by the Academy as to the danger of cycling. . . . . Meanwhile the Academy unanimously passed the following resolution: 'That the use of the bicyclette should only be permitted after a serious medical examination of the would-be rider.'" Now the least discerning person in the world would have said, "let the subject be fully discussed first, and then if any resolution be called for let it be passed." Moreover, no body of men with the faintest sense of humour would have passed a resolution at all of the kind said to have been passed by the French Academy. An evening paper scoffingly suggests that its true inner significance is "One louis a visit, if you please." One might as well suggest that everybody who proposes to eat pork should first be medically examined, as that every man, woman, and child cyclist should consult a doctor before he can be permitted to cycle. This is physic run mad with a vengeance if the *Times*' telegram be true. We cannot help hoping, however, even yet, that Dalziel's Agency has been the subject of a practical joke. But if not, we hope the French Academy of Medicine will pass no more resolutions about cycling, at any rate without first submitting them for revisal to a jury of English schoolgirls.

### The Asylum of the World.

A Polish emigrant, who landed at Hull the other day, had evidently a high notion of British wealth and charity. He had no money, he knew no English, yet he came to our shores in faith, nothing wavering. The shelter of the workhouse he refused scornfully; he wanted something more than that. He desired to be taught a trade, at English public expense, and meanwhile to receive a weekly allowance from the same source. The request was, in its way, a flattering testimonial to England's reputation throughout the world, but it was hardly to be gratified on that account, and it is to be feared that our Pole will have to find his way to fortune by the same rough roads that our own children go. Admitting, as we have begun to admit, that it is our national duty to provide technical education to as great an extent as we can, we have yet a divine recommendation to "begin at Jerusalem." The accident of birth, of which we talk so lightly and so scornfully, is a God-arranged accident nevertheless, and those of our kin—of our own land, have the first claim upon us. One would not willingly see Britain lose her premier position as the asylum of the oppressed, but the oppressed should bring with them some other stock-in-trade besides empty hands and a hungry mouth. When the Huguenot refugees came to Spitalfields they brought with them a trade which they could successfully practise and teach, and England never has been slow to welcome the stranger who could do something beyond the power of her own children and teach them a new art. But we have not room nor bread for our own incompetent, and still less for those of other lands. If they come to our shores and ask for our money, we are justified in asking them what work they are prepared to give in exchange. There is no more room for the non-worker in Britain than elsewhere. The wealth of England, of which so much is said, is created and maintained by labour; let an indefinite number of non-labourers enter the hive and the store of honey will soon become insufficient. Let Britain be the asylum of the nations; but she cannot afford to be the poorhouse of the world.

